# Neonatal Citrulline Supplementation and Later Exposure to a High Fructose Diet in Rats Born with a Low Birth Weight: A Preliminary Report

**DOI:** 10.3390/nu9040375

**Published:** 2017-04-11

**Authors:** Nhat-Thang Tran, Marie-Cécile Alexandre-Gouabau, Anthony Pagniez, Khadija Ouguerram, Clair-Yves Boquien, Norbert Winer, Dominique Darmaun

**Affiliations:** 1 INRA, UMR 1280, Physiology of Nutritional Adaptations, University of Nantes, IMAD and CRNH-Ouest, Nantes 44000, France; thang.tranobs@gmail.com (N.-T.T.); marie-cecile.alexandre-gouabau@univ-nantes.fr (M.-C.A.-G.); anthony.pagniez@univ-nantes.fr (A.P.); khadija.ouguerram@univ-nantes.fr (K.O.); clair-yves.boquien@univ-nantes.fr (C.-Y.B.); norbert.winer@chu-nantes.fr (N.W.); 2 Department of Gynecology and Obstetrics, Centre Hospitalier Universitaire Hotel-Dieu, Nantes 44000, France; 3 Department of Gynecology and Obstetrics, University of Medicine and Pharmacy, Ho Chi Minh City 70000, Vietnam; 4 Nutrition Support Team, IMAD, University Medical Center of Nantes, Nantes 44000, France

**Keywords:** low birth weight, amino acids, metabolic syndrome, liver, lipidomics, developmental origins of health and disease (DOHaD)

## Abstract

A low birth weight (LBW) leads to a higher risk of metabolic syndrome in adulthood. Literature suggests that citrulline supplementation in adulthood prevents the effect of a high fructose diet on energy metabolism. Whether neonatal citrulline supplementation would alter early growth or energy metabolism in the long-term in rats with LBW is unknown. LBW pups born from dams fed a low (4%) protein diet, were nursed by normally-fed dams and received isonitrogenous supplements of either l-citrulline or l-alanine by gavage from the sixth day of life until weaning, and were subsequently exposed to 10%-fructose in drinking water from weaning to 90 days of age. The oral glucose tolerance was tested (OGTT) at 70 days of age, and rats were sacrificed at 90 days of age. Pre-weaning citrulline supplementation failed to alter the growth trajectory, OGTT, plasma triglycerides, or fat mass accretion in adulthood; yet, it was associated with increased liver triglycerides, decreased liver total cholesterol, and a distinct liver lipidomic profile that may result in a predisposition to liver disease. We conclude that pre-weaning supplementation with citrulline does not impact early growth, but might impact liver fat metabolism in adulthood upon exposure to a high fructose diet.

## 1. Introduction

Being born with a low birth weight (LBW), whether due to an intra-uterine growth restriction (weight <10th percentile for gestational age) or to preterm birth, is not only a leading cause of perinatal mortality [1], but also a risk factor for the subsequent development of chronic disease such as obesity, type-2 diabetes, and cardiovascular disease in adulthood [2]. Moreover, neonatologists face a dilemma regarding the nutritional management of such infants. The detrimental effects of postnatal under-nutrition on brain development have long been documented [3], and largely explain why neonatologists aim at obtaining a rapid ‘catch up’ growth so that LBW infants reach an appropriate weight for their post-conceptional age at the time of hospital discharge. This goal has, however, often remained elusive; many preterm infants experience an “extra-uterine growth restriction”, between birth and hospital discharge [4]. On the other hand, data have emerged to suggest that excessive catch up growth, per se, may produce deleterious effects in the long run [5]. The search for specific nutrients that would enhance early postnatal growth without exposing individuals to a deleterious metabolic outcome in adulthood is therefore quite relevant for the nutritional management of LBW infants. 

An excessive dietary fructose intake has long been suspected to favor body fat accretion in humans. A recent meta-analysis concluded that prolonged exposure to a high fructose intake is associated with the elevation of fasting plasma glucose and triglycerides, as well as blood pressure, in humans [6]. Accordingly, a high fructose diet is associated with increased lipogenesis, insulin resistance, and hypertension in adult rats [7].

In the last decade, arginine, a semi-essential amino acid that is the sole endogenous precursor of nitric oxide (NO), was shown to impact both protein and energy metabolism. Arginine supplementation was found to prevent excess weight gain and improve glucose tolerance in genetically obese rats or rats fed a Western diet [8,9], presumably through effects mediated via the activation of AMPK [9,10]. Enhanced endothelial nitric-oxide synthase expression attenuated hypertension and hyperinsulinemia in fructose-fed rats [11]. The inhibition of arginase, the main enzyme accounting for arginine disposal, alleviated the hypertension produced by a high fructose intake in rats [12], and long-term oral l-arginine administration increased peripheral and hepatic insulin sensitivity in humans with type-2 diabetes [13]. 

Citrulline, a non-essential amino acid that is not incorporated into protein, is produced in the small intestine [14]. Contrary to arginine, citrulline, whether endogenous or exogenous, escapes splanchnic uptake, and is taken up by kidney, where it is converted to arginine [15]. Accordingly, oral citrulline was shown to be an attractive alternative to arginine, since oral citrulline enhanced the arginine bioavailability in both adult humans [16] and pregnant rats [17]. Moreover, recent work suggests that citrulline supplementation may prevent liver fat accretion in a model of non-alcoholic fatty acid liver disease (NFALD) induced by either a high fructose intake [18] or Western diet [19] in adult rodents.

In recent studies, we found that oral citrulline supplementation during gestation enhanced fetal growth and protein anabolism in a model of intrauterine growth restriction (IUGR) induced by dietary protein restriction in pregnant rats [20,21]. To the best of our knowledge, whether postnatal citrulline supplementation impacts growth or glucose metabolism in IUGR is not known.

We hypothesized that neonatal citrulline supplementation would enhance growth and prevent the metabolic consequences of exposure to excess fructose in later life. As our purpose was to determine whether citrulline would improve postnatal growth in pups that had suffered intra-uterine growth restriction, only LBW pups were studied. Therefore, the objective of the current study was to determine whether early, postnatal oral citrulline supplementation would alter: (1) early growth; and (2) fat mass accretion and glucose tolerance in adulthood, when rats born with LBW were exposed to a high fructose diet after weaning.

## 2. Materials and Methods 

### 2.1. Experimental Design

**Figure 1:**
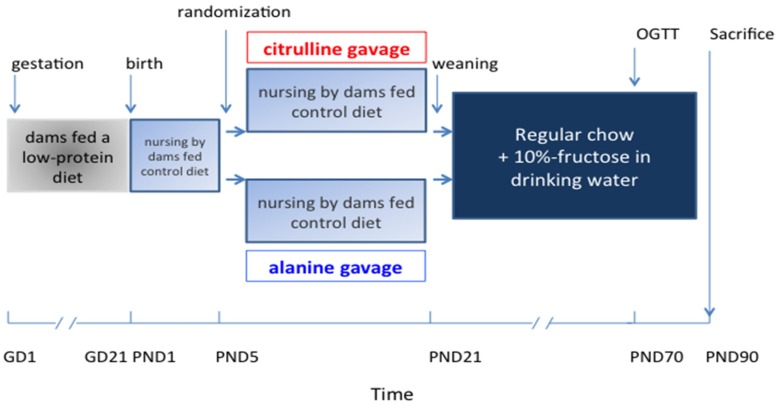
Protocol design. OGTT: Oral Glucose Tolerance Test; PND: Post Natal Day, G: gestation day.

All procedures were carried out in accordance with current institutional guidelines on animal experimentation in France and were approved by the Animal Ethics Committee of Pays de La Loire (Protocol number CEEA.2010.8). Female Sprague-Dawley rats weighing 200–250 g were purchased from Janvier (Le Genest Saint Isle, France), and delivered to our facility on gestational day one (GD1). After the first day of acclimation, they were randomized to receive either a standard diet (NP; *n =* 4), or a low protein (LP; *n =* 8) chow diet containing 4% protein (Figure 1). Dams were housed individually in a room kept at a constant temperature of 22 ± 1 °C with a fixed 12 h light–dark cycle, and were allowed free access to the experimental chow and drinking water during the entire gestation. Pups were not weighed immediately at birth (PND1) to prevent maternal stress, which could lead to rejection, or even cannibalism. On day 2 (PND2), pups born to NP dams and female pups born to LP dams were discarded, and male pups born to LP mothers were fostered by the NP mothers until weaning, with a standardized litter size of 8-pups per dam. The male offspring were also randomly assigned to two groups (*n =* 14/group), to receive, by oral daily gavage, either l-Citrulline (2 g/kg/day) or an isonitrogenous amount of l-Alanine (3 g/kg/day), starting on day six of their life and continued until weaning. Gavage was performed using PE 50 tubes with a length of 30 mm, ID of 0.58 mm (0.023 inch), and OD of 0.965 mm (0.038 inch). 

After weaning, rats were housed in individual cages, and drinking water was replaced for eight weeks with a 10% fructose solution (*w*/*v*) in both groups. Fresh solution was prepared daily and drinking containers were cleaned every other day. Food and fluid intake were estimated daily, from the amounts of food and water remaining in the trough and drinking bottles after 24 h, respectively, assuming minimal spilling. The body weight of the rats was recorded every week. At 70 days of age (PND70), rats were submitted to an oral glucose tolerance test. At the end of the study (90 days of age-PND90), rats were sacrificed by cervical dislocation. Liver and adipose tissue were dissected and stored. 

### 2.2. Biochemical Measures

Plasma hepatic transaminases, triglycerides, and HLD cholesterol levels were determined by using appropriate enzymatic assay kits (Roche Cobas^®^, Roche Diagnostics France, Meylan, France). Plasma insulin was measured using a kit purchased from Millipore^®^ (Millipore, Molsheim, France), according to manufacturer’s instructions.

Liver triglyceride and total cholesterol concentrations were determined using a DiaSys kit (Diagnostic System, Grabels, France), following a preliminary organic phase extraction as described in [22]. Briefly, 50 µg of liver samples were crushed with 500 µL of 150 mmol/L sodium chloride. Then, 150 µL of liver homogenates were extracted with 600 µL of a methanol-chloroform mixture (1:1, *v*/*v*). The organic layers were collected after centrifugation (10,000× *g* for 10 min) and dried under nitrogen. Dry samples were reconstituted in 37.5 µL of isopropanol/acetonitrile/water mixture (2:1:1, *v*:*v*:*v*) and 10 µL were analyzed, according to the manufacturer’s recommendations.

### 2.3. Non-Targeted Liver Phenotyping by Using Liquid Chromatography-High Resolution-Mass Spectrometry 

Liver samples of PND90 rats were extracted as described above. Dried organic layers were reconstituted in 400 µL of isopropanol/acetonitrile/water mixture and then 10 µL were injected into the Liquid Chromatography-Mass Spectrometry (LC-MS) system (a Waters Acquity H-Class^®^ UPLC-Synapt G2 HDMS) on an Acquity UPLC CSH C18 1.7 μm, 100 × 2.1 mm reverse-phase column (Waters Corp., Milford, MA, USA). Electrospray ionization (ESI) was used in a *m*/*z* range from 100 to 1200, in both positive and negative modes. Lipidomic data were processed, using the open-source XCMS^®^ [23], and all of the (*m*/*z*; RT) features were manually checked for the quality of their integration on the liquid chromatogram and their validity in quality controls (CV < 30%). Among 6701 (*m*/*z*; RT) features detected in the positive mode and 3741 in the negative mode , only 2747 and 671 features, respectively, met both of the criteria cited above. An annotation of the generated lipidomic profiles and subsequent identification of putative biomarkers of interest were achieved using an in-house reference databank [24].

### 2.4. RNA Isolation and Real-Time Polymerase Chain ReactionRT-PCR

The total RNA was extracted from aliquots of ~30-mg samples of frozen liver in PND90 rats using an RNA isolation kit (NucleoSpin RNA^®^, Macherey-Nagel, Germany), according to manufacturer’s instructions. The mRNA of genes involved in: (1) fatty acid and triglyceride synthesis (fatty acid synthase (FAS) and acetyl-coA carboxylase (ACC)); di- and tri-acylglycerol (acyl-CoA:diacylglycerol acyltransferases (DGAT1 or DGAT2)), with triacylglycerol synthesized by DGAT1 being preferentially channelled towards oxidation, whereas DGAT2 synthesizes triacylglycerol destined for very low-density lipoprotein assembly; unsaturated fatty acids (stearoyl-CoA desaturase (SCD1)) or cholesterol synthesis (3-hydroxy-methyl-glutaryl-CoA-synthase (HMGc1) and HMG-CoA reductase (HMGcr)); and triglyceride secretion (microsomal TG transfer protein, (Mttp)); (2) hepatic cholesterol degradation to bile acids (cholesterol 7alpha-hydroxylase (Cyp7a)), in β-oxidation (carnitine palmitoyltransferase 1a (Cpt1a)); and (3) transcription factors involved in the regulation of lipid synthesis (sterol regulatory element-binding transcription (Srebf1)) or lipid oxidation (peroxisome proliferator-activated receptor (PPARα)), were quantified by real-time RT-PCR. Primers were designed on the basis of the sequences available at the National Center for Biology Information gene bank using the PerlPrimer^®^ program (v.1.1.17, http://perlprimer.sourceforge.net) and synthesized by Eurogentec (San Diego, CA, USA). mRNA expression was calculated as previously described [25].

### 2.5. Oral Glucose Tolerance Test

At PND70, plasma insulin and glucose concentrations were measured in response to an oral glucose load. On the day before the test (at 9 p.m.), food was removed from all groups and fructose solution was replaced with regular drinking water. On the morning of the test (at 10 a.m.), a baseline blood sample (0 min) was drawn from the tail vein. Each animal then received an oral glucose dose of 1 g/kg, administered as a 50% (weight/volume) solution by oral gavage. Blood samples were collected at 15, 30, 60, 90, and 120 min after the oral glucose load and Accu-check^®^ (Millipore, Molsheim, France) was used to determine the blood glucose concentration. Peripheral IS index (ISI) during OGTT (ISI_0,120_)—an index that was found to be tightly correlated with the insulin sensitivity index determined using the hyperinsulinemic, euglycemic clamp [26]—was estimated using the equation: ISI_0,120_ = *m*/*MPG*/log(*MSI*), where *MPG* is the mean plasma glucose concentration (mg/L, mean of the 0 and 120 glucose values from the OGTT), *MSI* is the mean serum insulin concentration (mU/L, mean of the 0 and 120 insulin values from the OGTT), and *m* is the glucose uptake rate (mg/min), calculated as follows: glucose load (mg) + (0 min glucose − 120 min glucose, mg/L) × 0.19 (glucose space, L) × BW (body weight, kg)/120 min, as previously described in humans [26] and rats [27]. The area under the curve (AUC) of blood glucose was calculated using Graphpad Prism^®^ (GraphPad Software, Inc., San Diego, CA, USA).

### 2.6. Statistical Analysis

Values are expressed as means ± SEM (standard error of the mean). Differences among nutritional groups were analyzed by the non-parametric Mann-Whitney U-test using Graphpad Prism^®^, software version 6.0, and a value of *p* < 0.05 was considered as a significant difference between groups. Principal components analysis (PCA) of LC/MS data was performed using SIMCA^®^ software package (version 13.0, Umetrics, Umea, Sweden), in order to visualize any groupings of the generated data set and identify potential atypical or outlier individual data. The susceptibility of hepatic lipidomic phenotypes to citrulline supplementation was assessed by using a supervised method, Partial Least Squares Discriminant Analysis (PLS-DA). These PLS-DA models were applied to point out the variables with a major influence on the cluster membership. The quality and robustness of the PLS-DA were evaluated by several goodness-of-fit parameters and criteria, including: R2 (*X*), the proportion of the total variance of the dependent variables that is explained by the model; R2 (*Y*), defining the proportion of the total variance of the response variable (i.e., the class of the samples) explained by the model; and *Q*2, a seven-round internal cross-validation of the data reflecting the goodness of prediction of the model. A good prediction model is achieved when *Q*2 > 0.5, and if *Q*2 > 0.9, it is regarded as displaying an excellent predictive ability. The variable importance to the projection (VIP) values (VIP above 1.0), by using multivariate PLS-DA, were used to select discriminating metabolites in liver lipidome associated with citrulline supplementation.

## 3. Results

### 3.1. Energy Intake

**Figure 2:**
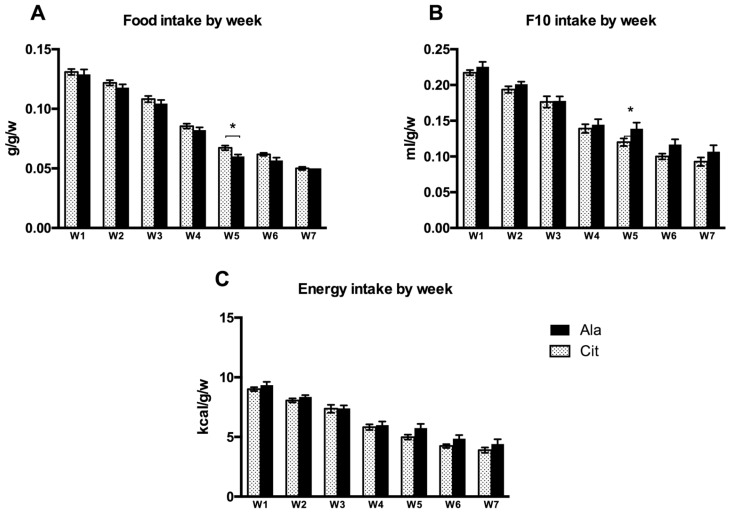
Post-weaning, weekly food intake (g/g body weight/week; (**A**)), consumption of 10%-fructose water (F10; mL/g body weight/week; (**B**)), and overall energy intake (kcal/g body weight/week; (**C**)) in rats that had received either citrulline or alanine by gavage in the pre-weaning period. W1: week 1, W2: week 2, and so on. Data represents means ± SEM (standard error of the mean) * *p* < 0.05 Cit vs. Ala rats (Mann-Whitney U test).

The post-weaning energy intake was estimated based on the daily food and fluid intakes and the caloric values of rat chow (2.8 kcal/g, A03 formula purchased from Safe^®^ (Safe Villemoisson-sur-Orge, France) and fructose (4 kcal/g), and did not differ between the Cit and Ala groups (Figure 2).

### 3.2. Growth and Body Composition

**Figure 3:**
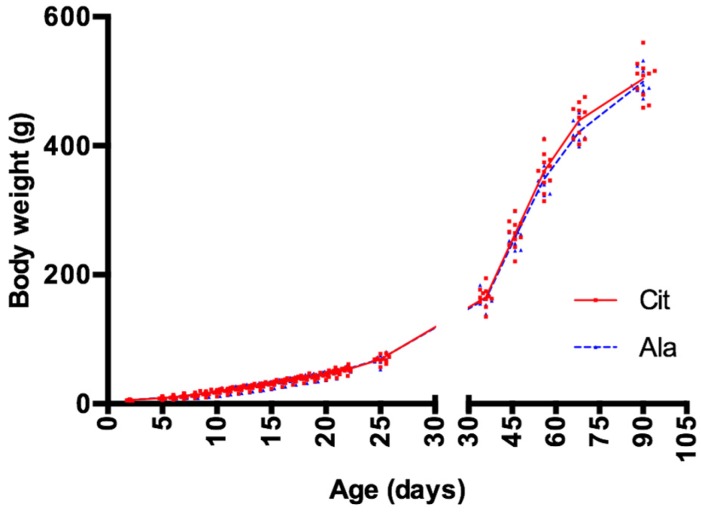
Body weight in rats born with intrauterine growth ertsriction (IUGR) prior to weaning while they received daily gavage with either citrulline (Cit) or alanine (Ala) (PND6–PND21), and after weaning while both groups received fructose supplementation in drinking water (PND21–PND90). IUGR: intrauterine growth restriction, PND: postnatal day.

No significant difference in body weight was observed between the groups, either during nursing or during fructose supplementation (Figure 3). Neither the fat mass nor the lean body mass differed between groups at the time of sacrifice (Table 1).

### 3.3. Glucose Metabolism (Figure 4)

During OGTT, the baseline blood glucose concentrations were comparable in both groups. Blood glucose rose higher in rats from the Cit group at 15 and 30 min after the glucose load, but returned to values indistinguishable from the Ala group at 60, 90, and 120 min; the area under the curve (AUC) did not differ between groups. The insulin sensitivity index, calculated from the blood glucose and serum insulin concentrations at 0 and 120 min, did not differ.

### 3.4. Lipid Metabolism

**Figure 5:**
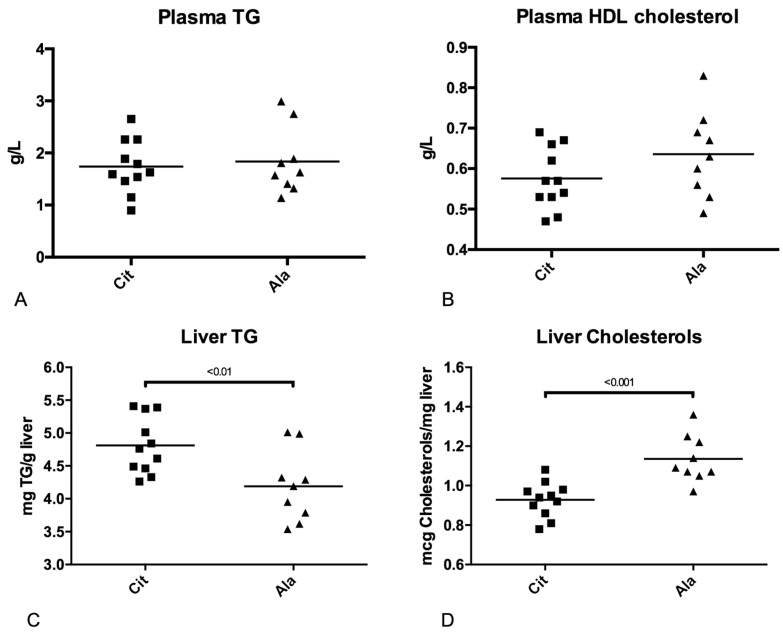
Plasma concentrations of triglycerides (**A**) and high-density lipoprotein (HDL)-cholesterol (**B**); liver triglyceride (**C**) and total cholesterol (**D**) concentrations in adult rats born with IUGR, pre-weaning supplemented with citrulline (Cit) or alanine (Ala), and receiving fructose supplementation in drinking water after weaning (Mann-Whitney U test).

The plasma TG and HDL cholesterol concentrations were similar in the two groups. In contrast, in adult rats that had received citrulline in early life, the liver TG concentration was higher (*p* < 0.01), whereas the total cholesterol level was lower (*p* < 0.001) than in rats that had received alanine (Figure 5). 

**Figure 6:**
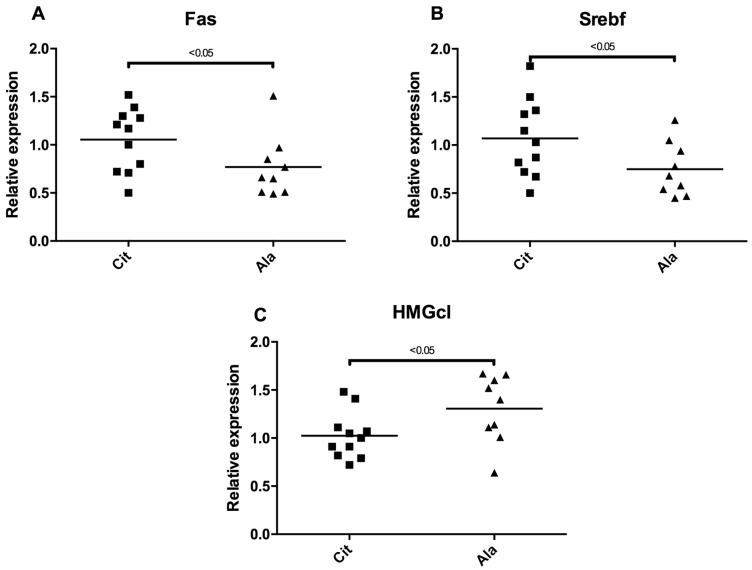
Relative expression level of genes ((**A**) Fas, fatty acid synthase; (**B**) Srebf, sterol regulatory element-binding transcription; (**C**) HMGc1, 3-hydroxy-methyl-glutaryl-CoA-synthase) involved in liver lipid synthesis between the citrulline (Cit) or alanine (Ala) groups.

Accordingly, the transcription of liver Fas and Srebf1, two genes involved in fatty acid synthesis, was elevated, and the transcription of HMGc1, an enzyme involved in the cholesterol synthesis pathway, was lower in the citrulline group, compared with the alanine group (Figure 6). The expression of DGAT1, DGAT2, SCD1, MTTP, HMG-CoA reductase, ACC, CPT1m and CYP 7A1, did not differ between the groups (data not shown).

**Figure 7:**
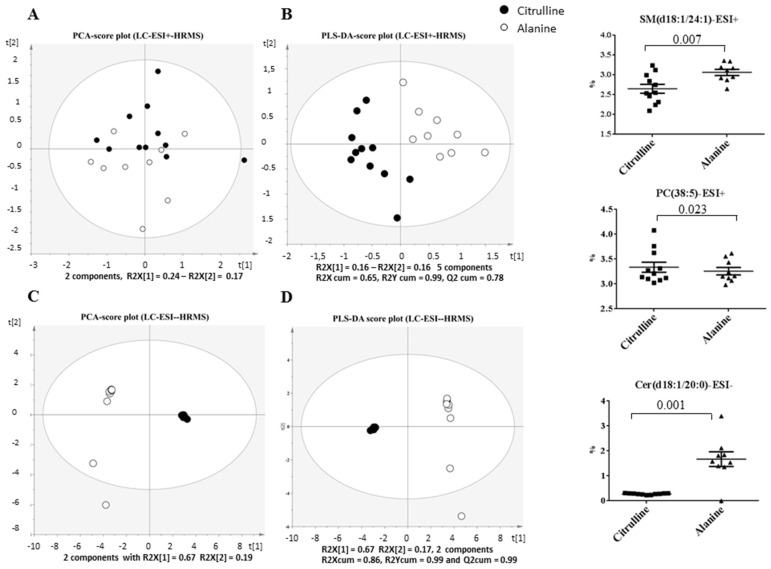
Principal Component Analysis (PCA) and Partial Least Square-Discriminant Analysis (PLS-DA) scatter plots of statistical models built on liver lipidomic profiles using Liquid Chromatography-Electro Spray Ionization positive (ESI^+^) (**A**,**B**) or negative (ESI^−^) (**C**,**D**) combined with High Resolution Mass Spectrometry (LC-ESI-HRMS) in adult rats born with Intra-Uterine Growth Restriction (IUGR) and receiving fructose supplementation in drinking water after weaning; effect of pre-weaning supplementation with Citrulline (Cit) or Alanine (Ala).

An exploratory principal component analysis (PCA) of the LC-MS lipidomics data was performed in positive and negative modes. The score plot of the first two PCs, which expressed almost 41% and 86% of the total variability for LC-MS profiles acquired in the positive (Figure 7A) and negative (Figure 7C) modes, respectively, showed a clear differentiation, particularly in the negative mode, between the Cit and Ala groups, reflected by a high goodness-of-fit and predictability, as indicated by an R2 value of 0.86 and by a Q2 value of 0.79, in the negative mode (Figure 7C). PLS-DA confirmed an equally good clustering of the samples with a high estimated goodness of prediction (Q2 around 85% and 99% for the first 2 components in the positive (Figure 7B) and negative (Figure 7D) mode, respectively, for the effect of early citrulline supplementation on adult lipodomic profiles (Figure 7B,C). The analysis of the corresponding loading plot revealed, among 1073 variables (i.e., (*m*/*z*; RT) features), 71 features that could be annotated with our home database [22], including 42 variables of importance for the clustering (VIP above 1.0) (Table 2). More specifically, among the 42 annotated VIP selected for the discrimination of Cit and Ala groups in PLS-DA, 16 VIP presented a significant mean discrepancy between the two groups, with specificities in several phospholipids (Table 2). Indeed, we observed a significant decrease in the Cit group in phosphatidylcholines containing saturated palmitic acid (i.e., PC (16:0/16:0) and PC (16:0/18:1)), but an increase in PC (18:2/20:3). In addition, the Cit group presented a significant enhancement in several phosphatidylethanolamines rich in arachidonic acid or its precursor, such as PE (16:0/20:4), PE (18:0/18:2), PE (18:0/20:4), and PE (18:2/20:4), but a trend towards a decrease in PE containing DHA as a fatty acid (i.e., PE (16:0/22:6), PE (18:0/22:6) and PE (18:1/22:6)). Furthermore, the Cit group had higher levels of hepatic phosphatidylinositol PI (16:0/18:2) and phosphatidylserine PS (18:0/20:4), but lower levels of one very long-chain sphingomyelin, SM (d18:1/24:1), and one long-chain ceramide, Cer (d(18:1/20:0), whereas no significant change was observed in the total triglycerides and diglycerides that could have been annotated, as compared with the Ala group (Table 2, Figure 7).

## 4. Discussion

### 4.1. Oral Citrulline Administration during Lactation Period Did Not Alter Pre-Weaning Growth

To the best of our knowledge, the current study is the first to address the effect of early post-natal citrulline supplementation on pup growth in low birth weight rodents. The lack of an anabolic effect of citrulline in the current study contrasts with the enhanced fetal growth observed with the antenatal maternal citrulline supplementation in our previous reports [20,21]. As citrulline may exert its effect through citrulline conversion to arginine, our finding also contrasts with the anabolic effect of postnatal arginine in neonatal piglets [28,29]. Inter-specific differences between rats and pigs may play a role. Alternatively, the anabolic effect of arginine supplementation was observed in piglets receiving artificial feeding, whereas pups were nursed and received natural milk from their foster mother in the current study. Moreover, low birth weight pups were adopted by normally nourished foster dams in the current study: nursing by normally-fed dams likely produced catch-up growth before PND6, regardless of the supplement administered by oral gavage between day 6 and 22.

### 4.2. Oral Citrulline Administration before Weaning May Impact Liver Lipid Metabolism in the Long Run

Very few studies have explored the long-term effect of supplementation with arginine or citrulline in early life. Tain et al. showed that maternal citrulline supplementation prevents the long-term alterations of renal function and blood pressure in offspring prenatally exposed to maternal energy restriction, dexamethasone, or L-NAME [30,31,32]. In the latter studies, citrulline supplementation was, however, given to mothers during gestation and lactation, rather than directly to pups, and no analysis was performed in pups either at birth, or at weaning. The specific time period (gestation or lactation) when citrulline exerted its effects in Tain’s studies therefore remains uncertain. In separate preliminary experiments, we found very low concentrations of citrulline in the milk of lactating dams receiving citrulline supplementation at 2 g/kg/day (192 µmol/L vs. 100 µmol/L in unsupplemented dams). Besides, the putative effect of citrulline on other aspects of metabolic syndrome, e.g., glucose tolerance and lipid metabolism, was not addressed in the latter studies.

In the current study, neonatal citrulline supplementation affected neither glucose tolerance, nor body fat mass or plasma triglyceride concentration, but increased the liver TG content. From a theoretical standpoint, the increased TG content could arise from increased lipogenesis, decreased β-oxidation, impaired TG export from the liver, or a combination of several of these mechanisms. We found an increased expression of genes involved in lipogenesis (FAS and its transcriptional regulator Srebf1) in the liver of the Cit group. In contrast, the expression of liver MTTP, a gene involved in TG secretion, was unaltered. Ceramides, synthesized from serine and palmitate, are known to stimulate TG secretion [33]. Ceramides and palmitate-rich phosphatidylcholines were lower in the liver of the Cit group. This suggests that palmitate availability may be limiting for ceramide synthesis in that group [34]. ACC converts acetyl-coA to malonyl-coA, which is a donor of 2-carbon moieties for fatty acid synthesis. Malonyl-coA, however, plays yet another role as a potent inhibitor of the entry of fatty acids into mitochondrion, and, therefore, inhibits β-oxidation [35]. Although we did not measure liver malonyl-coA concentration, ACC expression was unaltered in the Cit group compared with the Ala group (data not shown). We therefore speculate that the malonyl-coA concentration was likely unaltered in the Cit group, implying that β-oxidation was unaffected. Accordingly, CPT1 expression did not differ between the Cit and Ala groups (data not shown). Alterations in the fatty acid β-oxidation pathway therefore could not account for the hepatic TG accumulation in the Cit group. Taken together, our findings suggest that the liver fat accumulation observed in adulthood in Cit rats may be due, not to a decreased fatty acid oxidation or TG export, but to increased liver fat synthesis.

We observed a decrease in the liver cholesterol content in citrulline supplemented rats. Recent studies suggest that citrulline supplementation in adulthood may have a synergistic effect with statins in obese mice [36], and supplementation with watermelon juice (a citrulline-rich food) lowered LDL-cholesterol in adult hypercholesterolemic patients with MTHFR polymorphism [37]. We are not aware of any earlier study on the effect of early life citrulline on adult cholesterol metabolism. In the current study, the expression of the HMG-CoA reductase gene, the key enzyme in cholesterol synthesis, was unaltered in Cit rats. However, we observed a lower expression of HMGc1, a gene involved in the production of 3-hydroxy-3-methylglutaryl, an intermediate in the cholesterol synthesis pathway. Such a depletion of liver cholesterol is another piece of evidence suggesting an imprinting effect of early life citrulline supplementation on cholesterol metabolism after a high fructose challenge. 

Our data on the TG liver accumulation following citrulline supplementation contrast with studies in adult rats: fructose supplementation led to non-alcoholic fat liver disease with a significantly higher visceral fat mass, lower lean body mass, insulin resistance, and increased plasma triglycerides, and such effects were corrected by both non-essential amino acids and citrulline supplementation [18]. Similarly, Jegatheesan et al. [38] fed 200-g rats for four weeks with a 60% fructose diet, with or without 0.15 g/day citrulline (~0.75 g/kg/day). They observed that fructose supplementation produced liver steatosis and an elevation of plasma triglycerides, without altering glucose tolerance. Citrulline supplementation prevented hypertriglyceridemia and attenuated liver fat accumulation [38]. These authors showed that citrulline supplementation, when provided during adulthood, significantly decreased liver Srebf and Fas gene expression, without altering Mttp, ACC, and CPT1 gene expression, in rats fed with a 60% fructose diet [38]. Such genes were unaltered in the Cit group in the current study. Differences in the dose and timing of supplementation could account for such discrepancy. A higher dose of fructose (60% of overall intake) was given in earlier studies, so that protein intake likely decreased in the fructose-supplemented rats. In the current study, rats were fed an adequate protein diet, along with 10% fructose in drinking water, so as to mimic the consumption of a normal diet associated with soft drinks in humans. Secondly, we only supplied citrulline or alanine before weaning, as the rationale was to detect a long-term, rather than immediate, effect of citrulline supplementation. Thirdly, as non-essential amino acids were shown to mimic some of the metabolic effects of citrulline, the alanine-enriched drinking water used in the current study may produce effects similar to those of citrulline. In the current study, our purpose was to detect any effect that would be specific for citrulline, rather than a non-specific effect of the nitrogen supply; alanine was therefore chosen as a “placebo” because it allowed for an isonitrogenous placebo, and alanine failed to affect the growth or fat mass in studies by Jobgen et al. [9]. In retrospect, including an additional group without any amino acid supplement in early life would have been optimal.

Liver lipidomics were able to clearly discriminate, in adulthood, between rats that had received citrulline in early life, compared with those that had received alanine. We observed a decrease in the long chain Cer (18:1/20:0) assumed to be associated with insulin resistance, but also a decrease in the very long-chain sphingomyelin SM (d18:1/24:1) that is presumably associated with increased insulin sensitivity [39]. We observed no clear evidence of impaired glucose tolerance in the OGTT. Besides, the hepatic content in several long-chain PS and PE phospholipid species rich in arachidonic acid (AA; 20:4; *n*-6) or rich in its precursor, linoleic acid (18:2; *n*-6), was increased. Arachidonic acid is involved as a signaling molecule in multiple pathways, including inflammation [40] and the endocannabinoid system [41,42]. Our data suggest that citrulline supplementation before weaning may increase the arachidonic acid content and impact the cell membrane phospholipid composition in the liver of LBW rats subsequently fed a high fructose diet in adulthood. 

Whether such alterations would have a long term physiological impact is unknown. In a similar model of intrauterine growth restriction produced by restricting the protein content of maternal diet to 8%, rats who demonstrated catch-up growth at weaning displayed evidence for “metabolic inflexibility” by 200 days of age, which predisposes to metabolic syndrome later on in life [27]. Studies by other groups consistently showed that feeding dams an 8% protein diet (compared with a control, 20% protein diet) resulted in hyperinsulinemia and diabetes once offspring reached old age e.g., [43]. As a pilot study, the current study did not extend the observation of IUGR-born rats until old age. Such a question clearly requires further study.

## 5. Conclusions

In summary, the current preliminary report suggests that post-natal pre-weaning supplementation with citrulline does not impact growth, fat accretion, or glucose tolerance in rats born with a low birth weight and exposed to a high fructose diet after weaning. Our findings nevertheless suggest that neonatal citrulline administration may have an “imprinting” effect on liver lipidome and fat metabolism. The specific molecular mechanisms involved, and the potential impact of pre-weaning citrulline supplementation on liver lipid metabolism in adulthood, clearly warrant further exploration.

## Figures and Tables

**Figure 4:**
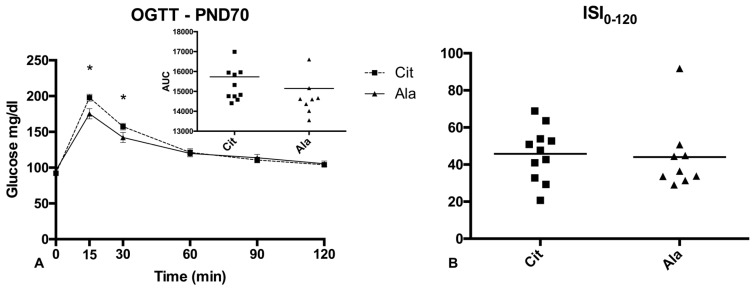
(**A**) Oral Glucose Tolerance Test (OGTT) performed at PND70 in rats born with IUGR and receiving fructose supplementation in drinking water after weaning; effect of pre-weaning supplementation with citrulline (Cit) or alanine (Ala) on blood glucose (means ± SD) and OGTT area under the curve (AUC; insert). (**B**) Insulin sensitivity index (ISI-0-120). * *p* < 0.05 Cit vs. Ala rats (Mann-Whitney U test).

**Table 1:** Body fat, lean body mass, and liver weight at the end of the study (PND90).

Tissue	Citrulline Group (*n =* 11)	Alanine Group (*n =* 9)	
	(mean ± SEM)	(mean ± SEM)	
White adipose tissue (WAT) (intra-abdominal)	21.19 ± 1.4 g	21.63 ± 1.8 g	NS
Intra-abdominal WAT/total WAT ratio	0.67 ± 0.02	0.67 ± 0.02	NS
Estimated lean body mass/body weight ratio	0.94 ± 0.00	0.94 ± 0.00	NS
Liver mass/body weight ratio	0.035 ± 0.001	0.036 ± 0.002	NS

NS: non-significantly different between alanine and citrulline groups.

**Table 2:** List of liver metabolites of interest in the discrimination of HPLC-ESI-HRMS-based lipidomic profiles on in both the positive and negative ionization mode and presenting a significant difference (*t* test on features mean, expressed as % of lipidomic profiles, with significant *p*-value < 0.05) between the citrulline and alanine group at PND90.

Lipid Class	Mode	Marker	Adduct	Citrulline Mean	Alanine Mean	*p*-Value—Unpaired *t* Test with Welch’s Correction
Phosphatidylcholines	ESI^+^	PC(32:0) PC(16:0/16:0)	(M + H)^+^	2.885	3.266	0.0444
	ESI^+^	PC(34:1) PC(16:0/18:1)	(M + H)^+^	4.8434	5.3756	0.0137
	ESI^+^	PC(38:5) PC(18:2/20:3)	(M + H)^+^	0.0574	0.0342	0.0227
Phosphatidylethanolamines	ESI^−^	PE(36:2) PE(18:0/18:2)	(M – H)^−^	3.6840	0.0000	<0.0001
	ESI^−^	PE(36:4) PE(16:0/20:4)	(M − H)^−^	11.4000	0.0000	<0.0001
	ESI^−^	PE(36:4) PE(18:1/18:3)	(M − H)^−^	1.8010	0.0000	<0.0001
	ESI^−^	PE(38:4) PE(18:0/20:4)	(M − H)^−^	13.6400	0.0000	<0.0001
	ESI^+^	PE (38:5) PE(18:1/20:4)	(M + H)^+^	1.3860	1.5560	0.0098
	ESI^−^	PE(38:6) PE(18:2/20:4)	(M − H)^−^	0.5036	0.0000	<0.0001
	ESI^+^	PE(38:6) PE(16:0/22:6)	(M + H)^+^	0.8097	0.8858	*0.0575*
	ESI^+^	PE(40:6) PE(18:0/22:6)	(M + H)^+^	0.4043	0.4734	0.0043
	ESI^+^	PE(40:6) PE(20:2/20:4)	(M + H)^+^	0.0136	0.0202	0.0085
	ESI^+^	PE(40:7) PE(18:1/22:6)	(M + H)^+^	0.1834	0.2133	*0.0624*
Phosphatidylinositol	ESI^−^	PI(34:2) PI(16:0/18:2)	(M − H)^−^	1.4680	0.0000	<0.0001
Phosphatidylserine	ESI^−^	PS(38:4) PS(18:0/20:4)	(M − H)^−^	5.8520	0.0000	<0.0001
Sphingomyeline	ESI^+^	SM(d18:1/24:1)	(M + H)^+^	2.6510	3.0650	0.0075
Ceramides	ESI^−^	Cer(d(18:1/20:0)	(M + HC − O_2_)^−^	0.2765	1.670	<0.0001
